# Department of Gynecology

**Published:** 1894-01

**Authors:** 

**Affiliations:** F. R. C. S., Ed., Professor of Anatomy University of Texas; late Physician for Diseases of Women, Edinburgh Providence Dispensary; Galveston


### Current Medical Literature.

### DEPARTMENT OF GYNECOLOGY.

EDITED BY WM. KEILLER, F. R. C. S., ED.,
Professor of Anatomy University of Texas; late Physician for Diseases of Women, Edinburgh Providence Dispensary.

HYSTERECTOMY — INDICATIONS AND TECHNIQUE. —(*J. M. Baldy*, *Am. Jour. Obstet. and Dis. of Women*, November, 1893.)

INDICATIONS. 1. *Malignant Disease.*—Indications absolute, complete removal only advisable operation, and justifiable if only one per cent. are cured. The earlier it is removed the better the result. Author has had three deaths in twenty-five operations, all of them preventable, and occurring in his earlier operations.

2. *Fibroid Growths.*—If tumor is small, slow growing, and giving no serious trouble, or if patient be not subject to conditions rendering inflammation probable, or be near the menopause, excision is unnecessary; but in all other cases operative treatment is indicated, and hysterectomy is the proper procedure. Myomectomy is to be preferred where it would be possible, and is desirable, that the patient should bear children. Ooporectomy he disapproves of, as often unsuccessful, often very difficult or impossible, and leaving behind a useless and diseased uterus.

3. *Pelvic Inflammations.*—The removal of diseased appendages is often unsuccessful, as regards cure of the patient, the uterus being still diseased, and the symptoms unrelieved. In all cases where the uterine walls are infiltrated with pus and the organ materially enlarged, hysterectomy is to be preferred to removal of the appendages.

4. *Prolapsus Uteri.*—Where other means have failed, where the prolapse is complete and of long standing, and the patient is at or near the menopause, the operation is applicable. It is particularly indicated where the uterus is much enlarged and the discharges and hemorrhages profuse. Such uteri are often cancerous.

5. *Inversio Uteri.*—In old chronic cases where taxis and elastic pressure have failed.

*Methods.*—In prolapse, inversion, malignant disease with small uterus, and small fibroids vaginal extirpation.

For fibroid tumors, large mnlignant uteri, and all inflammatory uteri, the abdominal operation is preferable. He prefers strong catgut ligature to clamps, and drops the stump back into the pelvis in preference to treating it extraperitoneally.

Except in malignant growths, supravaginal amputation of the uterus does all that can be done by total extirpation, and is less difficult, less tedious, less liable to sepsis, and has a smaller death rate.

The operation is always difficult, and should never be undertaken by an incompetent operator.

(The technique of each operation is very lucidly and very simply explained, and well illustrated; but cannot be condensed sufficiently.)

---

DR. EDEBOHLS, in the same journal, describes very clearly and shortly a bloodless method of removiug the fibromatous uterus. He curettes the cavity, and packs it and the vagina with sublimate gauze, before opening the abdomen. His paper is to be commended to those interested in the operation, but could not be intelligibly condensed.

---

VAGINAL HYSTERECTOMY.—(*E. E. Montgomery, M. D., Philadelphia, Ibid.*)

Dr. Montgomery condemns supravaginal amputation of the cervix for malignant disease of the uterus, and advocates total extirpation in all cases.

*Indications.*— Hysterectomy should be undertaken wherever the whole of the disease can be removed. This is not always easy to determine, but where there is a doubt, operate. Hemorrhagic fibroids, diseased appendages, and marked prolapse, may also call for removal of the uterus. The tendency of patients to ascribe irregular hemorrhages to the approaching menopause, and of physicians to prescribe ergot for hemorrhage without proper examination, is to be blamed for the fact that so many cases are beyond the possibility of relief before they apply for special advice. Malignant disease is found in young, as well as in old women.

*Symptoms.*—A thin, watery, odorous discharge, or irregular hemorrhage, is usually the first symptom, Pain may be absent, or appear late. The cervix is most often attacked; if it reveal nothing, the body should be explored, and a piece removed for microscopic examination.

Dr. Montgomery uses the Greig-Smith clamps, prefering them for:

1 Ease of application and economy of time.

2. Greater security against hemorrhage.

3. No chance of infection by ligature.

4. The thorough drainage secured by the clamps combined with a packing of gauze between them.

Remove clamps in twenty-four hours.

The technique is well described.

*Results.*—Twenty-one operations; one death, on fourteenth day, from tetanus.

Sixteen cases in which cancer was demonstrated by microscope. Nine in good health, of whom one was operated on five years ago, one nearly four years ago, one over three years, and the rest from four months to two years since. Seven have died since operation, the cancer manifesting itself again, usually within two months, and death taking place in from three to twelve months.

Five operations for bleeding fibroids; one death since, from cancer of mamma; rest in good health.

---

THE EXTRAPERITONEAL TREATMENT OF THE STUMP IN SUPRAVAGINAL HYSTERECTOMY.—(*Jos. Price, M. D.*, *Am. Jour. Obstet. and Dis. of Women*, November, 1893.)

Dr. Price strongly advocates the serre-neud and extraperitoneal treatment of the stump as having stood the test of long experience; while he states that those who condemn it, have no experience of the method itself, and only a limited experience of the intraperitoneal method.

In 111 cases of hysterectomy, for all kinds of diseases, he has never failed to apply the wire, and he states that it is applicable in all cases. Suppuration and fetor do not follow its use; it is a process of continual constriction and desiccation, without odor or oozing. No danger of trapping the bowel follows.

It is seldom possible to tie all the vessels in the intraperitoneal method, and hence oozing and the necessity for a drainage tube; with the serre-neud there is no oozing, and no drainage tube is needed.

The stump is frequently so inflamed, swollen, and œdematous, that it shrinks very much in the first few hours after operation. This is readily met by frequent tightening of the serre-neud, but cannot be met where the pedicle is treated intraperitoneally. It is in this particular that the uterine differs from the ovarian stump.

No ligatures or sutures are required where the neud is used; in the intraperitoneal method many ligatures and sutures are necessary, and each one of these is a posible source of danger.

One of the first elements of success in abdominal surgery is rapid operating—the extraperitoneal method is rapid, simple and safe; the intraperitoneal method slow, and complicated.

In the intraperitoneal method there is always a risk of septic peritonitis through the cervical canal—with the serre-neud this does not exist.

If for any reason the neud cannot be applied, the uterus should be completely extirpated, but this is a graver operation than the extraperitoneal treatment of a cervical stump.

---

PAN-AMERICAN MEDICAL CONGRESS. SECTION ON OBSTETRICS.

*Maternity Hospitals and Their Results.*—Dr. Jos. Price attributed the excellent statistics at modern maternities to the efficiency of the staff—cleanliness—absence of over crowding—and absence of tendency to hasten delivery. Mortality from sepsis has been reduced from 20% to almost nothing, and this was due to noninterference in normal labor, prompt aid when required, and strict cleanliness and hygiene.

*Axis-Traction Forceps.*—Dr. Hoffman showed a modification of Tarnier's instrument where the rods were attached to the blades by tapes. The movements of flexion and rotation of the fœtal head were not interfered with. The perinæum protected by its use.

Dr. Garigues commenting on Dr. Hoffman's paper approved of the principle of the tapes. He used the Simpson-Tarnier model. Did not think they saved the perinæum, but with them a mere tyro could do what it required a skilled obstetrician to do with the older instruments.

The power required was a mere fraction of that necessary formerly. He emphasized the importance of antiseptics in midwifery. No one should examine a woman without an antiseptic finger.

Dr. Jos. Price indorsed the axis-traction forceps. The use of other instruments was ofter equivalent to criminal assault.

The Simpson axis-traction forceps was a life-saving instrument. Many children could be delivered alive thus that with the older instruments would be still-born. He emphasized the importance of the antepartum vaginal douche as a means of guarding against ophthalmia neonatorum.

*Unqualified Midwifery.*—The section on obstetrics of the Pan-American Medical Congress passed a resolution protesting against the irregular practice of obstetrics by midwives, and recommending that, in view of the great danger to the community incident thereto, the board of health or licensing boards of various States refuse to grant licenses to applicants to practice midwifery who have not received technical instruction and preliminary training for at least one year in competent schools, and passed an examination in obstetrics equal to that required of applicants for the degree of medicine.

